# Lysine nutrition in swine and the related monogastric animals: muscle protein biosynthesis and beyond

**DOI:** 10.1186/s40064-015-0927-5

**Published:** 2015-03-27

**Authors:** Shengfa F Liao, Taiji Wang, Naresh Regmi

**Affiliations:** Department of Animal and Dairy Sciences, Mississippi State University, Mississippi State, MS 39762 USA

**Keywords:** Lysine, Muscle, Amino acid, Metabolism, Protein synthesis, Epigenetic regulation, Protein modification, Gene expression, Swine, Monogastric animal

## Abstract

Improving feed efficiency of pigs with dietary application of amino acids (AAs) is becoming increasingly important because this practice can not only secure the plasma AA supply for muscle growth but also protect the environment from nitrogen discharge with feces and urine. Lysine, the first limiting AA in typical swine diets, is a substrate for generating body proteins, peptides, and non-peptide molecules, while excess lysine is catabolized as an energy source. From a regulatory standpoint, lysine is at the top level in controlling AA metabolism, and lysine can also affect the metabolism of other nutrients. The effect of lysine on hormone production and activities is reflected by the change of plasma concentrations of insulin and insulin-like growth factor 1. Lysine residues in peptides are important sites for protein post-translational modification involved in epigenetic regulation of gene expression. An inborn error of a cationic AA transporter in humans can lead to a lysinuric protein intolerance condition. Dietary deficiency of lysine will impair animal immunity and elevate animal susceptibility to infectious diseases. Because lysine deficiency has negative impact on animal health and growth performance and it appears that dietary lysine is non-toxic even at a high dose of supplementation, nutritional emphasis should be put on lysine supplementation to avoid its deficiency rather than toxicity. Improvement of muscle growth of monogastric animals such as pigs via dietary lysine supply may be due to a greater increase in protein synthesis rather than a decrease in protein degradation. Nevertheless, the underlying metabolic and molecular mechanisms regarding lysine effect on muscle protein accretion merits further clarification. Future research undertaken to fully elucidate the metabolic and regulatory mechanisms of lysine nutrition could provide a sound scientific foundation necessary for developing novel nutritional strategies to enhance the muscle growth and development of meat animals.

## Introduction

Pork, the most consumed meat in the world, is one of the most economical sources of animal proteins for human consumption. Pigs grow fast, offer more meat per breeding female and, therefore, are more prolific than other livestock species (Adesehinwa et al. 2010). The goal of pig production is to convert feedstuffs into edible pork for high quality food proteins. The predominant component of pork is skeletal muscle (interchangeably called muscle in this review) and, in modern days, the efficiency of pork production is measured by the efficiency of lean (i.e., muscle) gain rather than whole body weight gain. Thus, the knowledge about the growth and development of muscle of pigs is fundamentally important from either a technical or an economic standpoint.

It has been known for decades that the growth and development of muscle of pigs essentially requires dietary supply of protein, or its components, amino acids (AAs), to be exact. There are about 20 AAs in nature (referred to as standard proteinogenic AAs) that serve as building blocks for protein biosynthesis, but not all AAs are indispensable dietary components because swine can *de novo* synthesize about 10 of them. Consequently, the essential dietary AAs are defined as those that need to be supplied exogenously because pigs cannot *de novo* synthesize them or cannot synthesize enough for their metabolic needs (Wang et al. 2014). Among these essential AAs, lysine is the first limiting one in swine nutrition management because it is the most deficient AA in nearly all typical swine diets based on cereal grains (Lewis 2001; NRC 2012). For this reason, lysine holds a very special, if not the paramount, significance in swine nutritional management practices. Some of the benefits of dietary lysine supplementation in swine and poultry production practices are summarized in Table 1.

**Table 1:** **Two major practices and the associated benefits of dietary lysine supplementation for swine and poultry** ^**a**^

***Practice***	**Benefits**
*To meet the lysine requirement with diets that have or have not met the crude protein requirements*	Making up for the lysine deficiency of feed ingredients
	Saving the cost on expensive feedstuffs of protein sources
	Reducing the energy need for deaminating excess AAs
	Maintaining or improving animal performance
	Maintaining or even increasing the production profits
*To further decrease the dietary concentration of crude protein*	Decreasing manure N concentration and in turn the N excretion to environment
	Further saving the cost on expensive feedstuffs of protein sources
	Reducing the energy losses associated with excess urinary N and heat increment
	Decreasing the NH_3_ emission into the air
	Reducing the odor in the production facilities
	Using inexpensive feedstuffs of alternative protein sources
	Decreasing animal water consumption
	Decreasing the volume of animal waste such as manure

^a^Data sourced from Kerr et al. (1995, 2003); Le Bellego et al. (2002); Otto et al. (2003); Guay et al. (2006).

Previous investigations have shown that dietary supplementation of crystalline lysine can improve muscle protein accretion and whole-body growth of pigs. In experiments with growing and finishing pigs, lysine supplementation increased the nitrogen retention and protein accretion, and improved the growth performance of the animals (Sharda et al. 1976; Fuller et al. 1987; Salter et al. 1990; Roy et al. 2000; Shelton et al. 2011). Furthermore, it has been suggested that the increase in muscle protein accretion was due to a greater increase in the rate of protein synthesis, rather than a greater decrease in the rate of protein degradation (Roy et al. 2000; Salter et al. 1990). Nevertheless, the underlying metabolic and molecular mechanisms by which dietary lysine regulates muscle mass accumulation of pigs is not clear (Wu 2010b; Rezaei et al. 2013). Thus, in this review, the up-to-date knowledge of lysine metabolic and physiological functions related to muscle growth and development of pigs is summarized. It needs to be pointed out that a large portion of the knowledge was appropriated from the research on other monogastric animals including humans because the swine-related research in this regard is very limited in the literature.

## Metabolic functions of lysine

### Biosynthesis of proteins and peptides

Like any other proteinogenic AAs, the major function of lysine in animal lives is to serve as one of the 20 types of building blocks for synthesis of body proteins and peptides, which are indispensable organic compounds participating in virtually all biochemical reactions and physiological activities (including structural support) of all living cells and tissues. Without lysine-involved protein and peptide syntheses, living cells or living animals could not exist.

Animal body proteins perform diverse biochemical and physiological functions necessary for life. Most of these proteins are physically presented as cellular or tissue constituents, such as those in the muscle. Functional proteins include enzymes, transporters, hormones, and antibodies, while reproductive proteins are those in sperm, eggs, and milk. The critical functions of these proteins are enormous and beyond the scope of this review.

A typical mammalian cell requires tens of thousands of different proteins and peptides at any given moment, and each of these proteins or peptides, like living organisms on the earth, has a life span. Due to this life span, a protein population within a cell has a constant turnover process where old or unneeded proteins are degraded and new proteins are *de novo* synthesized. The dynamics of protein turnover within a cell or tissue, however, is programmatically controlled by animal genetic makeup, but it can be regulated by various environmental factors including nutrients, such as AAs.

**Figure 1:**
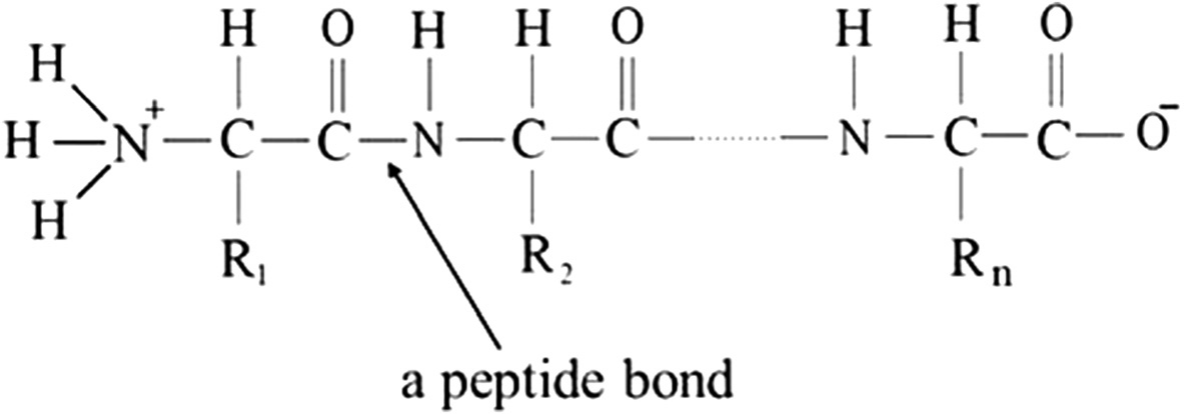
**A general formula of a peptide molecule.** This representation shows at least one peptide bond. R_1_, R_2_, and R_n_ represent side chains of n amino acid residues. With a loss of one molecule of water from two amino acids, one peptide bond (−CONH−) is formed. When n = 0 (i.e., the third amino acid residue does not exist), the peptide will be a dipeptide; when n = 1, the peptide will be a tripeptide; and so on.

Structurally, proteins are polymers of AA residues linearly connected by amide bonds, commonly known as peptide bonds. A peptide bond is a link between an α-carboxyl group of one AA to an α-amino group of another (Figure 1). Most natural polypeptides contain 50 to 2,000 AA residues in each molecule and are commonly referred to as proteins, while oligopeptides (short chain peptides) consist of only 2 to 30 AA residues. Beside the number of AA residues, the dividing line between proteins and peptides is also based on their molecular weights (5.5 to 8,000 daltons for oligopeptides). However, the line based on molecular weight is not absolute and, in some cases, the three-dimensional structure needs to be considered (Wu 2013a).

Parathyroid hormone (PTH), an example of polypeptide, is secreted by parathyroid glands. This hormone contains 84 AA residues. The main function of PTH is to regulate calcium and phosphate homeostasis and vitamin D synthesis within animal body. The lys-13 residue in PTH is very important for the folding of the active domain of the hormone (Zull et al. 1987), and it was found that a poly-L-lysine preparation can enhance the PTH-stimulated bone resorption (Raisz et al. 1979).

The oligopeptides, of which lysine is an integral part, also play important roles in animal bodies. For example, some poly-lysine-containing peptides (14 AA residues) are found to affect the activities of some membrane enzymes including protein kinases, phosphatidylinositol kinases, and adenylate cyclase (Gatica et al. 1987). An oligopeptide that contains 10 or less AA residues is usually called a small peptide. The shortest oligopeptides are dipeptides, consisting of only 2 AA residues joined by a single peptide bond, followed by tripeptides, tetrapeptides, etc. Examples of small peptides include kallidin (10 AA residues: Lys-Arg-Pro-Pro-Gly-Phe-Ser-Pro-Phe-Arg) and lysine-vasopressin (9 AA residues: Cys-Tyr-Phe-Gln-Asn-Cys-Pro-Lys-Gly). Kallidin and bradykinin are naturally generated bioactive peptides in body fluids and tissues through proteolytic cleavage, and they function as vasodilators for the maintenance of normal blood pressure (Wu 2013a; Lafarga and Hayes 2014). Like arginine-vasopressin (in other mammals), lysine-vasopressin (in pigs) is a peptide hormone that stimulates the reabsorption of water in the distal tubules of the kidney, leading to the formation of more concentrated urine (Nielsen et al. 1995).

For protein and peptide syntheses in an animal body, a pool of free AAs must be available simultaneously at the site of the syntheses. Free AAs normally result from the catabolism of dietary and body proteins. The efficiency of recycling body protein AAs for new protein synthesis, however, is far from 100%, and therefore, a large portion of free AAs must come from the intestinal digestion of dietary proteins. Failure to obtain enough AAs from diets will result in intense degradation of body proteins, especially the muscle proteins, because muscle is the largest, dynamic, body protein reservoir.

As other dietary AAs, the small intestinal absorption of free lysine is generally more rapid than the absorption of protein-bound lysine, and the rates of absorption of protein-bound AAs have been shown to vary and may be affected by the source of protein, its degree of processing, as well as the energy component of the diet (Leibholz et al. 1986). In pigs, the absorption of free lysine is complete by the end of ileum, and the concentration of plasma lysine reaches its peak 1 to 2 hours after feeding (Leibholz et al. 1986). From a human study (Uhe et al. 1992) it was found that it took 5 to 7 hours for dietary lysine to be transported into muscle tissue after ingestion. Compared to other essential AAs, free lysine is more concentrated in the intracellular space of muscle tissue, which suggested that muscle serves as a body reservoir for free lysine.

### Generation of non-peptide molecules

Besides its primary function as a building block for biosynthesis of proteins and peptides, lysine also functions as a substrate for generation of numerous non-peptide molecules, which include low molecular-weight nitrogenous substances (e. g., carnitine, polyamines, ammonia, and urea), other AAs or AA derivatives, as well as some non-nitrogenous small molecules (Wu 2013a). Each of these metabolites has specific biochemical and physiological importance for animal life processes.

Carnitine, synthesized from lysine and methionine via a multi-step biochemical process, is a quaternary ammonium compound that is required for transport of long-chain fatty acids from cytoplasm into mitochondria for β-oxidation, a major mechanism for ATP production in insulin-sensitive tissues such as skeletal muscle, heart, liver, and adipose tissue (Steiber et al. 2004). Besides a role in normalizing blood cholesterol and triglyceride concentrations, carnitine plays additional physiological roles in protecting organisms from oxidative stress, promoting substrate oxidation in brown adipose tissue, improving cardiac performance, and regulating energy partitioning in the body (Ferrari et al. 2004; Wu 2013a).

Hydroxylysine is synthesized from lysine by lysyl hydroxylase reaction called hydroxylation (Hausmann 1967). As is known, collagen is the most abundant family of proteins in the extracellular matrix of connective tissues which include skin, bone, cartilage, and tendon, while elastin is another major component of certain soft connective tissues, such as arterial walls and ligaments (Halper and Kjaer 2014). Both collagen and elastin are cross linked to form fibrous proteins based on aldehyde formation from the amine side chains of lysine or hydroxylysine residues, and it is lysyl oxidase, an enzyme, that converts the amine side chains of the lysine or hydroxylysine residues into aldehydes (Eyre et al. 1984). In addition, hydroxylysine also represents special sites for the attachment of carbohydrates in collagen (Gelse et al. 2003). Both collagen and elastin play very important roles for defining the structural integrity and physiological functions of the extracellular matrix of connective and muscle tissues (Purslow et al. 2012; Gelse et al. 2003; Wang et al. 2013).

Glutamate is the most significant excitatory neurotransmitter in the mammalian central nervous system, and lysine, present at high concentration in the brain, is an important precursor for *de novo* synthesis of glutamate. Papes et al. (2001) showed that the synthesis of glutamate from lysine, which is carried out by the saccharopine pathway (a lysine degradation pathway in mammals), is very likely to take place in neurons.

Cadaverine is a foul-smelling diamine compound (a type of polyamine with exactly two amino groups) produced by protein hydrolysis during putrefaction of animal tissue, and specifically it is synthesized from lysine in a one-step reaction with lysine decarboxylase (Andersson and Henningsson 1981). Although polyamines in general are recognized as cell growth factors in relation to cell proliferation, differentiation, regeneration, and malignant transformation, the specific physiological functions of cadaverine are not clear (Patocka and Kuehn 2000). Because it had an acute oral toxicity of 2,000 mg/kg body weight in rats (Til et al. 1997), a high level of cadaverine residue in muscle may affect meat hygienic quality for human consumption (Stadnik and Dolatowski 2010).

### Lysine catabolism and energy source

Like carbohydrates and lipids, AAs can also be used to meet animal energy requirement, especially when carbohydrates and lipids become unavailable to provide enough energy for animals. In three metabolic circumstances, free AAs undergo post-absorptive oxidation to provide the animal with energy: First, when a diet is rich in protein and the released AAs exceed the body’s needs for protein synthesis, the surplus AAs are oxidized to ammonia and carbon dioxide via the formation of keto acid that enters the tricarboxylic acid (TCA) cycle to produce energy. The glucogenic AAs can also provide energy through gluconeogenesis pathway. Secondly, as aforementioned, there is a constant protein turnover in virtually all living cells, and based on its own AA composition each protein requires a certain ratio of free AAs supplied for its synthesis. At a given time, the cellular free AA ratio usually does not exactly match the cellular requirements for syntheses of new proteins and, furthermore, most cells or tissues unfortunately do not have a mechanism to store free AAs. The “extra” free AAs can be metabolized to other biologically active substances or catabolized to produce energy according to the dynamic needs and metabolic potentials of the cells. Thirdly, during starvation or in uncontrolled diabetes mellitus, when carbohydrates are either unavailable or not properly utilized, cellular proteins, especially those from muscle tissue, will be utilized as fuel for the body.

AAs are an important and specifically required fuel for several tissues. After absorption, AA oxidation supports 15% of the resting energy expenditure of humans (Battezzati and Riso 2002). During feeding, the splanchnic bed extracts and immediately oxidizes a large amount of enteral non-essential AAs, including the totality of glutamate and the majority of glutamine and alanine (Battezzati and Riso 2002). Stoll et al. (1998) hypothesized that besides glutamine, lysine is an important energy source for the small intestine. During exercise, muscle AAs may produce significant amounts of energy via deamination of aspartate to provide ammonia for the synthesis of adenosine monophosphate (AMP) from inosine monophosphate (IMP) and intermediates for the TCA cycle (Battezzati and Riso 2002).

**Figure 2:**
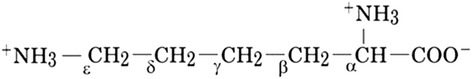
**A general formula of lysine.** Lysine is a cationic or basic amino acid with an α-amino group, a long side chain, and an ε-amino group. Presented in this formula is an ionized form of lysine.

As a truly essential AA, lysine is a cationic or basic AA with a long side chain (Figure 2), and its metabolism begins with the intestinal uptake from digesta mainly via a Na^+^-independent transport system. After absorption, the free lysine in excess of the needs for syntheses of proteins and other substances will be catabolized in a cell- and tissue-specific manner (Gatrell et al. 2013). The intestinal oxidation of enteral lysine contributed one-third of total body lysine oxidation in growing pigs fed a high-protein diet (van Goudoever et al. 2000). Other tissues such as liver, kidney, muscle, and brain also contribute to the whole body lysine catabolism.

**Figure 3:**
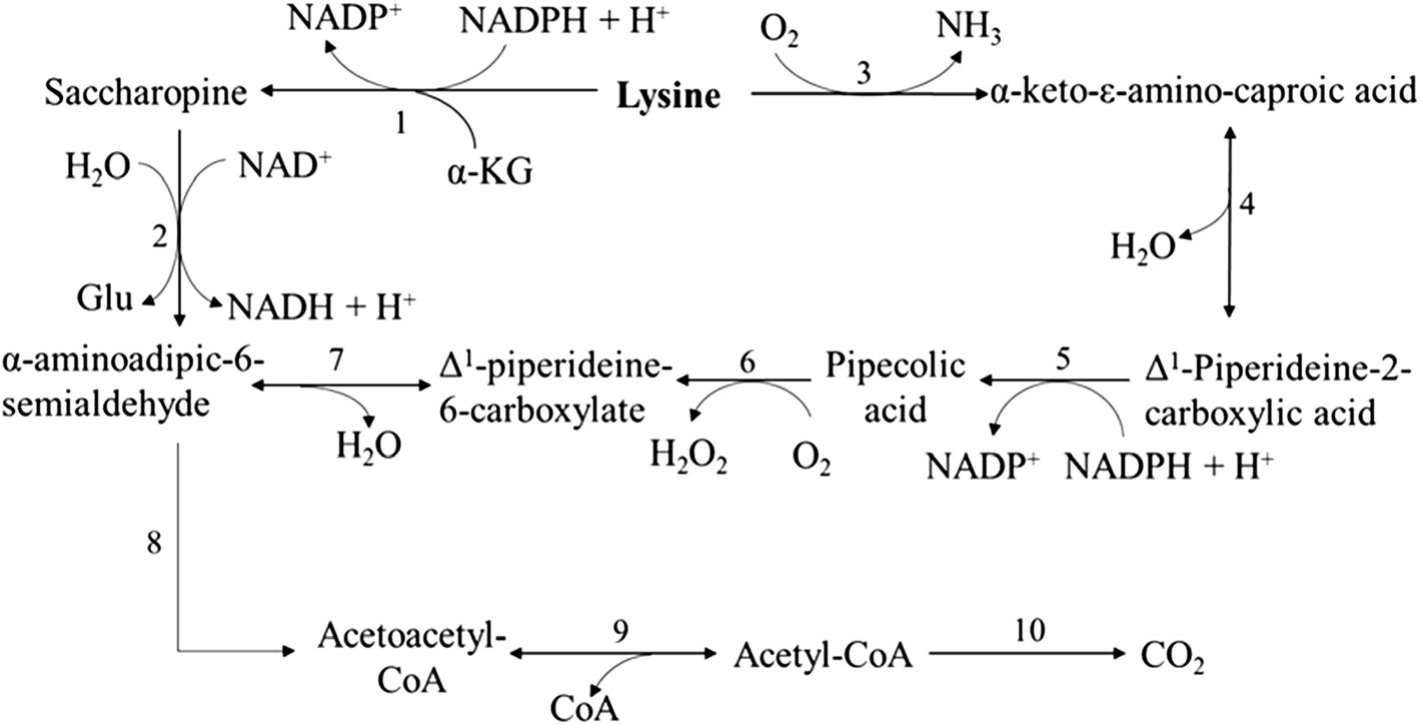
**Lysine catabolism in monogastric animals.** Lysine is catabolized via the saccharopine pathway and the pipecolate pathway. The enzymes involved include: (1) Lysine α-ketoglutarate reductase; (2) saccharopine dehydrogenase; (3) lysine oxidase; (4) spontaneous; (5) peperideine-2-carboxylic acid reductase; (6) pipecolate oxidase; (7) spontaneous; (8) enzymes including aminoadipate semialdehyde dehydrogenase, aminoadipate aminotransferase, α-ketoacid dehydrogenase, glutaryl-CoA dehydrogenase, glutaconyl-CoA decarboxylase, enol-CoA hydratase, and β-hydroxyacyl-CoA dehydrogenase for seven steps; (9) thiolase; (10) enzymes of the TCA cycle. Adapted from Wu (2013a).

The catabolism of lysine is very unique relative to the catabolism of other AAs in that it proceeds mainly through two distinct metabolic routes, the saccharopine pathway and the pipecolate pathway, both of which later converge into one common degradative pathway (Figure 3). These two pathways differ in that the saccharopine pathway is predominantly mitochondrial, whereas the pipecolate pathway is predominantly peroxisomal and cytosolic (Hallen et al. 2013).

The primary pathway of lysine catabolism is thought to be the saccharopine pathway in liver (Papes et al. 1999; Gatrell et al. 2013). In this pathway (Figure 3), lysine first combines with α-ketoglutarate (α-KG) to form an adduct, saccharopine, by the catalysis of lysine-ketoglutarate reductase (LKR). Then saccharopine is converted to α-aminoadipic-6-semialdehyde and glutamate by saccharopine dehydrogenase (SDH), which is a part of a single polypeptide, bifunctional aminoadipate δ-semialdehyde synthase (AASS) as LKR is (Gatrell et al. 2013). The α-aminoadipate-6-semialdehyde is subsequently converted into acetyl-CoA via a few more steps (Wu 2013a). This pathway is unusual in the way that the ε-amino group is transferred to α-KG and then into the general nitrogen pool. The further oxidation of acetyl-CoA produces CO_2_ and energy via TCA cycle.

Small portion of lysine are catabolized in the brain through pipecolate pathway (Chang 1976). In this pathway (Figure 3), the α-amino group rather than ε-amino group of lysine is removed during the conversion of lysine to pipecolate or pipecolic acid in cellular peroxisomes. The intermediates of this pathway include α-keto-ɛ-aminocaproic acid, Δ^1^-piperideine-2-carboxylic acid, and Δ^1^-piperideine-6-carboxylate (Broquist 1991; Wu 2013a). The ability of mammalian brain to synthesize pipecolic acid suggests a role as a neurotransmitter, and in pathological situations pipecolic acid accumulates in body fluid (Broquist 1991). In addition to the two pathways just discussed, there are some other undefined pathways that can also contribute to lysine catabolism, which include those depending on lysyl oxidase, L-AA oxidase, and carnitine biosynthesis (Benevenga and Blemings 2007; Gatrell et al. 2013).

While the amino groups of lysine are converted to ammonia, which is further converted to urea or uric acid through the urea cycle, the end product of the carbon skeleton catabolism is acetyl-CoA, which is further catabolized for energy via TCA cycle or converted to ketone bodies or fatty acids. Because acetyl-CoA is the fuel for the TCA cycle and cannot be converted to glucose by pigs and other mammals, lysine is strictly ketogenic in nature. The carbon atoms in ketone bodies are ultimately degraded to carbon dioxide via the TCA cycle to produce energy for the animal body (Berg et al. 2002).

Nonetheless, of all essential AAs, lysine is the most strongly conserved one, as demonstrated in rats and chicks (Flodin 1997; Benevenga and Blemings 2007). Meredith et al. (1986) reported that in young men when lysine intake was reduced, the oxidation of lysine decreased significantly, which was supported by the study of the activity of liver LKR (the initial enzyme in the saccharopine pathway) that was decreased in rats treated with less lysine (Chu and Hegsted 1976). These findings suggested that lysine is unique in that it is less catabolized than most, if not all, other essential AAs. This unique conservative nature of lysine is very interesting because lysine is the most deficient AA in almost all typical diets for monogastric animals such as pigs.

## Physiological functions of lysine

Beyond the metabolic functions described above, lysine also exerts many physiological functions for monogastric animals. Lysine can affect animal metabolism of other nutrients, hormone production, and immunity (Wu 2010a; Wu 2013b). More remarkably, peptide bound lysine is a potential active site of post-translational modification (PTM) and epigenetic regulation of gene expression. Understanding these physiological functions of lysine within the animal body is requisite for animal scientists and producers to better use lysine for promoting animal health and production (Wu 2010a).

### Lysine effect on plasma AA profile and nutrient metabolism

AAs are directly and indirectly related to each other within the overall nutrient metabolism pathways, and the plasma AA concentrations reflect the whole sum of the metabolic flow from all organs and tissues (Yen et al. 2004; Shikata et al. 2007). Interactions among AAs within an animal body alter the expected release of AAs into the blood from diet. Lysine-arginine antagonism is one of the classic examples of the interactions. Because lysine and arginine share some common chemical properties, excess dietary lysine increases the requirement for arginine by chicks (O’Dell and Savage 1966). Another example of AA interactions is the interaction among three branched-chain AAs (leucine, isoleucine and valine). Garcia-Villalobos et al. (2012) tried to explain some complexity of AA absorption and concentrations in plasma. They assumed that typical diets formulated to meet the requirement of lysine contain excess of leucine, which may depress the absorption of lysine causing depression of pig performance. Their results indicated that the dietary leucine to lysine ratio affects the expression of cationic AA transporters in jejunum and muscle, and therefore, affects plasma AA profile.

A study of plasma AA network structure in rats showed that a single AA deficiency can affect plasma AA profiles, for which lysine is located at the top control level, affects the metabolism of almost all other AAs, but is not influenced by others (Shikata et al. 2007). Zimmerman and Scott (1965) reported that dietary lysine deficiency resulted in increased plasma concentrations of some AAs, notably isoleucine, threonine and valine in chicks. Braude et al. (1974) performed a research trial with growing pigs fed diets based on cereals and groundnut meal and supplemented with graded amounts of lysine. They found that the concentration of lysine in blood plasma increased linearly over a wide range of lysine content, but the concentrations of most other AAs, however, were largely unaffected. Similarly, Roy et al. (2000) studied plasma AA profiles in growing barrows fed diets deficient, adequate or excess in lysine, and found that the plasma lysine concentration increased whereas the concentrations isoleucine, taurine, threonine and valine decreased, as the dietary lysine concentration increased. The plasma concentration of histidine decreased, and the concentration of serine increased in pigs fed either lysine deficient or excess diet. The plasma concentrations of all other AAs were not affected by the diets.

Besides on other AAs, dietary amount of lysine also has effects on the metabolism of other nutrients. Jarowski and Pytelewski (1975) supplemented a control diet with 0.114% lysine to rats and reported a decreased serum cholesterol level, and the cholesterol level was increased again when the rats were switched back to the control diet. Dietary lysine supplementation can regulate Ca metabolism, specifically enhancing intestinal Ca absorption and improving renal conservation of the absorbed Ca (Civitelli et al. 1992). The arginine-nitric oxide (NO) pathway can be modulated by elevated levels of lysine in endothelial cells because lysine is a natural inhibitor of arginine transport through shared y^+^ system (Liaudet et al. 1997). With lipopolysaccharide-treated neonatal pigs, Carter et al. (2004) reported that the NO synthesis in isolated lung was significantly inhibited by lysine perfusion.

In addition, lysine can form transitory complexes in various enzymes with cofactors including biotin, pyridoxal, and lipoate (Broquist 1991). With human liver tissue, Zhao et al. (2010) reported that virtually every enzyme in glycolysis, gluconeogenesis, TCA cycle, urea cycle, fatty acid metabolism, and glycogen metabolism can be acetylated, which revealed that lysine acetylation plays a major role in regulation of cellular metabolism in response to nutrient availability, cellular metabolic status, and extracellular conditions.

### Lysine effect on hormone production and activities

It has been recognized that insulin, growth hormone (GH), glucocorticoids, insulin-like growth factor 1 (IGF-1), thyroid hormones, and some other hormones regulate body protein and energy metabolism including muscle protein turnover (Breier 1999; Liu et al. 2006). Among these hormones, GH, IGF-1, and their associated carrier proteins and receptors work together as a multi-level hormonal system called the somatotropic axis (Breier 1999; Tomas et al. 1992), which has been regarded as a key regulatory pathway for muscle growth. Previous research in rats showed that the activity and function of this axis can be significantly affected by the nutritional status, such as plasma AA levels or dietary AA supply (Straus and Takemoto 1990; Takenaka et al. 2000).

Plasma IGF-1 concentration in rats fed a low-lysine (20% of requirement) diet was reduced by approximately 28% (Takenaka et al. 2000). In nursery pigs fed a low-lysine diet (0.7% lysine), the plasma IGF-1 level was 52% lower than that of pigs fed the control diet (1.15% lysine), although no difference in hepatic IGF-1 mRNA abundance was found between the two groups (Katsumata et al. 2002). In other studies with growing pigs, however, dietary lysine did not show influence on plasma GH and IGF-1 concentrations (Roy et al. 2000; Ren et al. 2007). Different lysine levels and different animal physiological status may explain the discrepancy of the aforementioned studies.

Due to the lysine-arginine antagonism, administration of these two AAs together may have some antagonism elimination effect. It was reported that oral administration of a combination of lysine (1.2 g) and arginine (1.2 g) to young and healthy male human volunteers provoked a release of GH and insulin to the blood (Isidori et al. 1981). However, oral administration of arginine (3 g) and lysine (3 g) in old men did not increase serum GH or IGF-1 concentration (Corpas et al. 1993). In primiparous sows, high lysine intake increased postprandial concentrations of insulin and IGF-1, in which the effect was from a combination of lysine and other AAs (Yang et al. 2000).

Insulin is secreted primarily in response to the elevated blood concentration of glucose. Other nutrient stimuli including AAs can also promote insulin secretion. Although there are large differences among AAs in their capacities to stimulate insulin secretion, intravenous administration of lysine significantly increased the plasma insulin level in adult human subjects (Floyd et al. 1966). Some recent studies also suggested that lysine has a stimulating effect on insulin secretion, but this effect may be in a dose dependent manner. Roy et al. (2000) reported that plasma insulin concentration tended to increase in growing barrows fed a high lysine diet (0.98 vs. 0.75 and 0.45%), while the plasma triiodothyronine (T_3_) concentration decreased when the high to the low lysine diet (0.98 vs. 0.45%) was compared. Similarly, Ren et al. (2007) reported that dietary level of total lysine at a concentration of 0.71, 0.95, or 1.20% did not show any influence on plasma insulin concentration of growing pigs. However, when the lysine level was further increased to 1.45%, the insulin concentration was significantly increased.

The interactions between lysine and the aforementioned hormones might lead to a modification of either translational or transcriptional, or both events of protein synthesis in pigs (Ren et al. 2007), and it has been known that dietary supplementation of lysine can significantly enhance pig growth and production performance (Shelton et al. 2011). Surprisingly, how dietary lysine at different levels affect the activities of insulin and somatotropic axis pathway has not been thoroughly studied.

### Lysine effect on protein modification and gene expression

Epigenetics is defined as the study of heritable changes in gene activity and expression that occur without alteration in DNA sequence, and it is known that these heritable changes are tightly regulated by two major molecular modifications, DNA methylation and histone modifications, amongst several others (Goldberg et al. 2007; Hung and Sellappan 2008). While DNA methylation refers to the addition of a methyl group to the cytosine or adenine nucleotides of DNA, histone modifications mean the chemical modifications of histone proteins. Recognized as a PTM, the multivalent modifications of histone core include methylation, acetylation, phosphorylation, deimination, ubiquitination, and sumoylation at the amino terminals (Peterson and Laniel 2004; Hou et al. 2008; Bannister and Kouzarides 2011). Histone modifications are very critical for regulating chromatin structure (the DNA molecules packaged by histone) and function, which can in turn affect many DNA-related processes, such as transcription, recombination, repair, replication, and chromosomal organization (Bannister and Kouzarides 2011). In recent years, more and more research has been conducted to reveal how dietary nutrients affect epigenetic events in humans (Cobiac 2007; Ho and Dashwood 2010).

The numerous functions of the lysine residue in histone are largely related to its side chain ε-amino group, which is a main target involved in methylation, acetylation, ubiquitination, sumoylation, succinylation, etc. Methylation and acetylation of lysine residues at histone tails are the two most common PTMs with distinct distributions along both euchromatin and heterochromatin (Hung and Sellappan 2008). Interestingly, unlike other modifications, the same lysine residue in histone can be methylated to different degrees to include mono-, di- or tri-methyl moieties, which has been characterized as the unique pattern associated with various effects on gene activities (Hung and Sellappan 2008; Lan and Shi 2009). Acetyllysine (or acetylated lysine) is an acetyl-derivative of lysine, and lysine acetylation regulates the histone binding to DNA in nucleosomes and thereby controls the expression of genes (Wu 2009; Zhao et al. 2010). While the ubiquitination of histone lysine plays an important role in gene transcriptional initiation and elongation, histone sumoylation is a modification related to ubiquitination and involves the covalent attachment of small ubiquitin-like modifier molecules to the lysine residues (Bannister and Kouzarides 2011). While methylation or acetylation of histone can either activate or repress gene expression, methylation can also occur in many non-histone proteins (Lan and Shi 2009) and acetylation can occur in numerous transcription factors, nuclear regulators, and various cytoplasmic proteins to regulate cell processing (Glozak et al. 2005; Yang and Seto 2008; Wu 2013a).

As just mentioned, lysine can also play roles in the PTM of other eukaryotic proteins, which is of great importance for regulating the synthesis, the structure formation, and/or the behavior or functions of many proteins (Walsh 2006). For example, lysine can directly participate in protein methylation (e. g., trimethyllysine in calmodulin), ubiquitination, and *O*-glycosylation (Roberts et al. 1986; van den Steen et al. 1998). Lysine acetylation is a conserved protein PTM that links acetyl-CoA metabolism and cellular signaling (Choudhary et al. 2014). Eukaryotic translation initiation factor 5A (eIF5A) needs to be activated when a moiety of spermidine (a 4-aminobutyl group) is transferred to the active site of lysine residue of eIF5A (Folk et al. 1980). Ubiquitination is another PTM where ubiquitin is attached to a substrate protein, which affects proteins in many ways such as leading protein degradation via the proteasome, changing protein location and activity, and promoting or preventing protein interactions (Schnell and Hicke 2003). This process most commonly involves binding the glycine of ubiquitin to a lysine residue on the substrate, forming an isopeptide bond (Pickart 2001). Succinylation is a PTM where a succinyl group is added to a lysine residue of a protein molecule, including histones (Zhang et al. 2010).

It has been reported that pigs fed a low lysine diet grew more slowly and less efficiently than those fed the control diet and had a lower plasma IGF-1 level. However, there was no change in the level of liver IGF-1 mRNA expression. This finding reported by Katsumata et al. (2002) suggested a potential post-transcriptional mechanism that underlies the lysine-affected IGF-I gene expression.

Animal (including human) life essentially is a set of gene expression processes. Although these processes are genetically pre-programmed, dietary nutrients are the driving force for these processes. Understanding lysine effect on gene expression is critical in that it can inspire animal scientists to develop novel nutritional strategies by using alternative, less expensive feed ingredients to regulate the expression of the genes that are associated with muscle growth. To this end, He et al. (2013) conducted a feeding trial with weaned pigs to evaluate the effect of dietary lysine on the mRNA expression of three cationic AA transporters. Their results showed that the abundance of b^0,+^AT, y^+^LAT1, and CAT1 mRNA in jejunum was significantly affected by dietary lysine intake. Morales et al. (2014) also conducted a feeding trial with growing pigs to evaluate the effect of dietary lysine on a few selected genes, and their results showed that the expression of myosin mRNA in semitendinosus muscle was highly correlated with the dietary lysine level (r = 0.87). The expression of CAT1 mRNA in jejunum mucosa was higher in the pigs fed lysine-deficient diet. However, the expression of b^0,+^AT mRNA in jejunum mucosa was not different between the pigs fed the lysine-deficient vs. lysine-adequate diets.

### Disorders associated with lysine metabolism

Serious metabolic disorders associated with lysine metabolism can be caused by the failure of the transport systems for intestinal absorption of lysine. As lysine is essential for protein synthesis, failure of its transport within an animal body can lead to diminished protein synthesis, which can cause severe hyperammonemia after a protein rich meal. Poor intestinal absorption, together with increased renal elimination, of lysine is observed in the human lysinuric protein intolerance condition, an inborn error of AA metabolism caused by a defect of intestinal and renal transporter protein, y^+^LAT1, encoded by SLC7A7 gene (Torrents et al. 1999). Because the transporter protein y^+^LAT1 is responsible for the intestinal absorption and renal reabsorption of cationic AAs including arginine, lysine, and ornithine, the concentrations of these three AAs are reduced in plasma but increased in urine. Similarly, the transport of lysine together with arginine and ornithine are defective in both intestine and kidney of cystinuria patients (Thier et al. 1965).

Genetic disorder in either of the first two reactions of the saccharopine pathway of lysine catabolism (Figure 3) can result in familial hyperlysinemia. Because these two reactions are catalyzed by the bifunctional enzyme AASS, defects can be found in either the N-terminal half harboring the LKR activity or the C-terminal half harboring the SDH activity (Dancis et al. 1979). The deficiency of α-ketoadipate dehydrogenase is the reason for human α-ketoadipic aciduria because the α-ketoadipate, an intermediate in the catabolism of lysine and tryptophan, cannot be converted into glutaryl-CoA (Wilson et al. 1975). These deficiencies are commonly observed in individuals who excrete large quantities of urinary lysine and some saccharopine.

### Deficiency and toxicity of lysine

In livestock industry, the deficiency of dietary lysine significantly affects the growth performance and carcass characteristics of meat animals. Lower dietary lysine concentration relative to the adequate concentration negatively affects animal growth performance with decreased average daily gain and increased feed to gain ratio (Roy et al. 2000; Takenaka et al. 2000; Bidner et al. 2004). What’s more, it was reported that lower levels of dietary lysine increased the backfat thickness (Bidner et al. 2004; Tous et al. 2014). Witte et al. (2000) reported that the intramuscular fat content was increased in pigs treated less lysine. There was also reductions in loin eye area of the pigs fed less lysine diet (Witte et al. 2000; Bidner et al. 2004). Goodband et al. (1990) reported that the ham weights decreased with decreasing lysine level within a certain range among somatotropin treated pigs.

The deficiency of dietary lysine also impairs animal immune function leading to increased susceptibility of animals (including humans) to infectious diseases (Datta et al. 2001; Li et al. 2007). Because lysine is required as a building block for protein synthesis, a deficiency of lysine limits the synthesis of animal immunity-related proteins that include antibodies and cytokines to exert the necessary immune function. With chickens, Chen et al. (2003) reported that inadequate lysine intake negatively affected both antibody response for humoral immune function and cell mediated immune function. For humans, oral ingestion of appropriate dose of L-lysine monohydrochloride showed evidence of decreasing the severity of symptoms associated with recurrences, and even evidence of decreasing the recurrence rate of herpes simplex (McCune et al. 1984; Griffith et al. 1987). The mechanism for this antiviral activity may be involved in the antagonistic relationship between lysine and arginine. Arginine deficiency suppressed herpes simplex virus replication in tissue culture, which means arginine is required for the replication of herpes simplex virus (Griffith et al. 1981). Ingestion of lysine would compete with arginine for entry into virus and inhibit arginase activity (Wu and Morris 1998).

Because lysine is an important precursor for the synthesis of glutamate, which is the most significant excitatory neurotransmitter in the mammalian central nervous system, deficiency of lysine may lead to mental and physical problems due to the reduced glutamate synthesis (Papes et al. 2001). In humans, besides slow growth, the symptoms or signs of lysine deficiency include fatigue, nausea, dizziness, anorexia, irritability, anemia, and reproductive disorders.

Oral L-lysine intake is well tolerated and the toxicity of lysine seldom develops in animals. In a lysine toxicity study, rats treated with a diet consisting of over 5.0% lysine did not show any changes in clinical signs, body weight, diet consumption, water intake, ophthalmology, organ weights, gross pathology, or the histological structure and function of kidney (Tsubuku et al. 2004). It seems that animal body sustains a wide arrange of plasma lysine concentration without demonstrating any side effects. That the oral intake of lysine has a high safety margin may be because of (1) slower entry into circulation, (2) induction of high LKR activity in the liver, (3) more time for egress of lysine from circulation into muscle for temporary storage, and/or (4) more time for kidneys to respond to the increasing plasma lysine level by acceleration of urinary lysine excretion (Flodin 1997).

Nevertheless, it is worth noting that different body conditions may respond differently to lysine. When plasma lysine concentration reached up to 1,700 μmol/L, some people still looked healthy and were incidentally detected with hyperlysinemia, while others were found with problems such as motor mental retardation, seizures, muscular hypotonia, and spasticity (Saudubray and Rabier 2007). Rajantie et al. (1980) reported that dietary lysine supplementation caused abdominal cramps, and transient diarrhea in lysinuric protein intolerance human patients. Hyperlysinemia in humans is a concomitant of many metabolic errors, including inborn errors of lysine catabolism, urea cycle disorders, pyruvate carboxylase deficiency, and organic acid disorders (Saudubray and Rabier 2007). In pigs, dietary lysine level of more than 3 or 4 times the basal level slightly decreased weight gain and feed intake, but no other adverse effects were displayed (Edmonds and Baker 1987). Readers can refer to Wu (2014) and Wu et al. (2014) for the newly recommended values of dietary requirements of lysine and other AAs by pigs and chickens.

## Conclusions and perspectives

Improving the efficiency of nutrient utilization by pigs and other monogastric animals with the dietary application of AAs is becoming increasingly important, simply because this practice can secure a balanced plasma AA supply for muscle protein synthesis while protecting the environment from excess nitrogen output from the animal. Lysine, a truly essential AA, is not only a building block for *de novo* syntheses of almost all proteins and many peptides, but also as a substrate for producing non-peptide molecules, in animal bodies. Excess lysine in animal bodies can be catabolized and used as an energy source, although this energy source is not significant from a nutrition standpoint because lysine is uniquely conservative in catabolism. Understanding the metabolic fate of ingested lysine will help animal scientists develop novel nutritional strategies to improve the efficiency of nutrient utilization for muscle growth.

From a regulatory standpoint, lysine is located at the top control level in affecting other AA metabolism. Lysine can also affect the metabolism of other nutrients such as Ca and cholesterol. The effect of dietary lysine on hormone production and activities is reflected by the change of plasma concentrations of insulin and IGF-1. Lysine residues in peptide chains are important sites for PTM, which is involved in histone modification and epigenetic regulation of gene expression. In addition to PTM, lysine can also be involved in the post-transcriptional stage of protein expression. Deficiency of dietary lysine will impair animal immunity and elevate animal susceptibility to infectious diseases. In humans, an inborn error of a cationic AA transporter can lead to a lysinuric protein intolerance condition.

Commercial feed-grade crystalline lysine was introduced to animal feed industry in the late 1980s (Wittmann and Becker 2007). Because lysine deficiency has negative impact on animal health and growth performance, and lysine appears to be non-toxic even at a high rate of dietary supplementation, animal nutritionists should put more emphasis on dietary lysine supplementation to avoid lysine deficiency rather than lysine toxicity. Dietary supplementation of crystalline lysine for monogastric meat animals can significantly increase body muscle protein accretion, which may be due to a greater increase in the rate of protein biosynthesis rather than that of protein degradation. Nevertheless, the underlying molecular mechanism regarding how the dietary lysine regulates muscle protein turnover and which cell signaling pathways are involved for the turnover are still waiting for further clarification. Research in the future should be conducted to fully elucidate the metabolic and regulatory mechanisms of lysine nutrition, and this elucidation will benefit not only in promoting animal industry but also in improving human body composition as well.

## Abbreviations

AA: Amino acid
AAs: Amino acids
AASS: Aminoadipate δ-semialdehyde synthase
AMP: Adenosine monophosphate
ATP: Adenosine triphosphate
CAT1: Cationic amino acid transporter 1
CoA: Coenzyme A
ECM: Extracellular matrix
eIF5A: Eukaryotic translation initiation factor 5A
GH: Growth hormone
HMG-CoA: β-Hydroxy-β-methyl-glutaryl-CoA
hr: Hour(s)
IGF-1: Insulin-like growth factor 1
IMP: Inosine monophosphate
LKR: Lysine-ketoglutarate reductase
Lys: lysine
NO: Nitric oxide
PTH: Parathyroid hormone
PTM: Post-translational modification
SDH: Saccharopine dehydrogenase
SLC7A7: Solute carrier family 7, subfamily A, member 7
T_3_: Triiodothyronine
TCA: Tricarboxylic acid
